# Respiratory Health Impacts of Outdoor Air Pollution and the Efficacy of Local Risk Communication in Quito, Ecuador

**DOI:** 10.3390/ijerph20146326

**Published:** 2023-07-08

**Authors:** Jiang Zhou, Laura Gladson, Valeria Díaz Suárez, Kevin Cromar

**Affiliations:** 1 Marron Institute of Urban Management, New York University, Brooklyn, NY 11201, USA; 2 Department of Environmental Medicine, New York University Grossman School of Medicine, New York, NY 10010, USA; 3 Secretaría de Ambiente del Distrito Metropolitano de Quito, Quito 170138, Ecuador; 4 Department of Population Health, New York University Grossman School of Medicine, New York, NY 10016, USA

**Keywords:** air pollution, global health, health communication, Latin America, respiratory tract diseases

## Abstract

Relatively few studies on the adverse health impacts of outdoor air pollution have been conducted in Latin American cities, whose pollutant mixtures and baseline health risks are distinct from North America, Europe, and Asia. This study evaluates respiratory morbidity risk associated with ambient air pollution in Quito, Ecuador, and specifically evaluates if the local air quality index accurately reflects population-level health risks. Poisson generalized linear models using air pollution, meteorological, and hospital admission data from 2014 to 2015 were run to quantify the associations of air pollutants and index values with respiratory outcomes in single- and multi-pollutant models. Significant associations were observed for increased respiratory hospital admissions and ambient concentrations of fine particulate matter (PM_2.5_), ozone (O_3_), nitrogen dioxide (NO_2_), and sulfur dioxide (SO_2_), although some of these associations were attenuated in two-pollutant models. Significant associations were also observed for index values, but these values were driven almost entirely by daily O_3_ concentrations. Modifications to index formulation to more fully incorporate the health risks of multiple pollutants, particularly for NO_2_, have the potential to greatly improve risk communication in Quito. This work also increases the equity of the existing global epidemiological literature by adding new air pollution health risk values from a highly understudied region of the world.

## 1. Introduction

According to the World Health Organization (WHO), air pollution (both household and outdoor) is the largest environmental threat to human health, associated with 7.4 million premature deaths every year. Low- and middle-income countries experience greater exposure to unhealthy levels of air pollution compared to the global average [1]. However, not all global regions experience the same concentrations or composition of outdoor air pollution. For example, the concentrations and mixtures of outdoor air pollution in Latin America might be distinct from North America, Europe, and Asia due to different natural and anthropogenic sources of air and different meteorological and topographic features. Similarly, the health response of the general public may be modified due to differences in baseline health conditions, cultural or socioeconomic differences affecting exposure pathways, or distinct genetic makeup. Unfortunately, there are few studies about air quality and health in Latin America to demonstrate any such distinctions [2,3].

**Figure 1:**
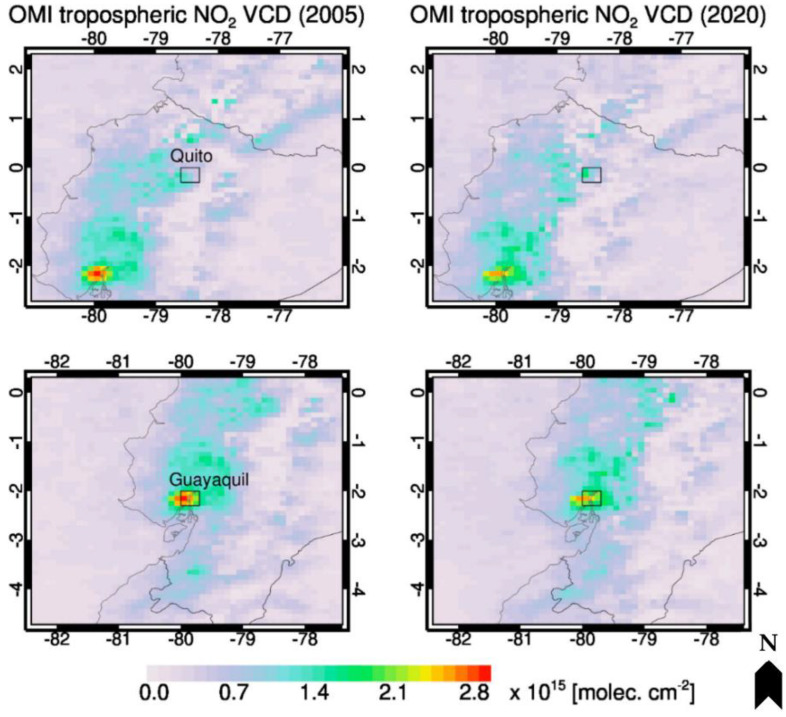
NO_2_ data trends for the cities of Quito and Guayaquil in Ecuador, 2005–2020 (OMI instrument, tropospheric NO_2_ vertical column density, 13 × 24 km^2^ horizontal spatial resolution). Reproduced with permission from Ref. [8].

This study will address these issues by performing a health analysis in Quito, the capital city of Ecuador, to determine local associations between air pollution and respiratory hospitalization data. Quito is a valley city surrounded by mountains, increasing the risk of temperature inversions, which, coupled with decades of fast population growth, have made the city highly susceptible to elevated air pollution episodes [4]. Vehicle emissions are of particular concern, driven by high-sulfur fuels and an increasing demand for private transportation [5]. However, recent air quality control efforts have resulted in improved air quality measured by local monitoring stations. In fact, the literature available suggests that air pollution levels may be lower in Latin America than in Europe, Asia, and North America. In some of the most polluted parts of Latin America, satellite-derived data demonstrate long-term trends of decreasing NO_2_ levels [6]. In Ecuador specifically, WHO’s Ambient Air Pollution in Cities database reports that Quito has relatively good air quality compared to other cities in the country and to other major cities in Latin America and around the world in terms of the annual mean concentration of fine particulate matter [7]. This is supported by data in Figure 1 showing satellite-derived NO_2_ data trends for the cities of Quito and Guayaquil in Ecuador from 2005 to 2020 [8]. While the concentrations of certain air pollutants in some major cities in South America have decreased since 2010, the region still consistently exceeds the WHO guidelines and national standards [9].

Many countries with varying levels of air quality choose to communicate current air pollution conditions to the public using air quality indices, which encourage individuals to modify their behavior in ways that reduce unhealthy air pollution exposures. Changes in behavior in response to index alerts have been observed in numerous studies [10,11,12]. However, traditional risk communication tools, such as the U.S. Air Quality Index (AQI), are designed to highlight days where pollution levels are above regulatory levels and are, therefore, limited in capturing the risks associated with lower levels of air pollution. As air quality has been improving in some regions, strong evidence suggests that even air pollution below standard regulatory levels is associated with increased health risk [13,14,15]. 

Efforts have been made in many countries to develop health-based indices for use as communication tools to the public [16,17,18,19]. Studies have evaluated these indices and found that health-based indices in general represent health outcomes more accurately than existing air quality indices [16,20,21]. Respiratory morbidity has improved through the awareness and utilization of the Air Quality Health Index (AQHI) in Canada [22], and a study in Shanghai found that an air quality health index, compared with the existing air pollution index (API), shows much stronger associations with health outcomes and therefore provides a more effective tool to communicate the air pollution-related health risks to the public [16]. Recently, Mexico City also created and validated a multi-pollutant, health-based air quality index, which is currently in use to communicate daily health risks to the public [23]. 

The Municipality of the Metropolitan District of Quito (MDMQ) has designed a numerical index, Quito’s Air Quality Index (IQCA), which is communicated to the public every day in order to guide individual behavior modification decisions and reduce the public health burden attributable to air pollution exposures. The IQCA is generated by converting the measured concentrations of air pollutants to a common numerical and color scale for all pollutants, with specific ranges tied to different impacts on human health. However, this index has never been evaluated for its ability to accurately capture the overall health risk to the Quito population. The need for a validation of air quality messaging using local health data has been recommended by leading experts at the American Thoracic Society [24], and directly informs the design of the present study.

The purpose of this work is two-fold: First, it evaluates the association between respiratory health risks and outdoor air pollution in Quito. Second, it assesses whether population-level respiratory health risks, the health outcome most likely to drive individual behavior modification decisions [25,26,27], are associated with the IQCA values communicated daily to those living in Quito. This work not only benefits Quito directly by providing location-specific risk values and communication improvements, but increases global health research equity by adding new air pollution health risk values to the limited epidemiological literature conducted in Latin America. 

## 2. Materials and Methods

### 2.1. Exposure Data

**Figure 2:**
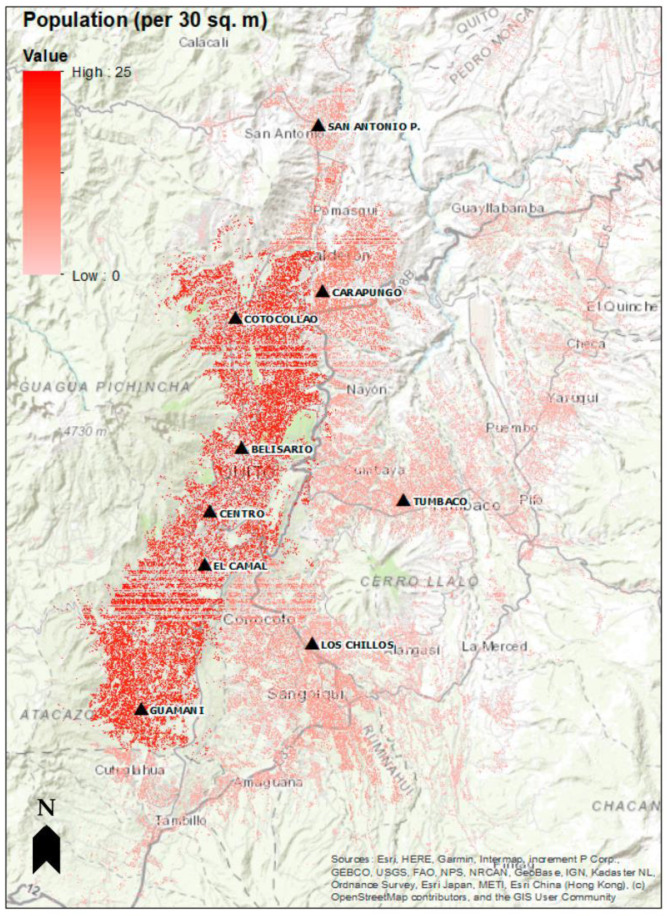
Locations of air pollution monitoring stations overlaid on population density in Quito. The black triangles represent the 9 REMMAQ stations: Belisario, Carapungo, Centro, Cotocollao, El Camal, Guamani, Los Chillos, San Antonio, and Tumbaco.

Hourly air pollution data in Quito for the years 2014–2015 were obtained for all 9 monitoring stations from Quito’s Atmospheric Monitoring Metropolitan Network (REMMAQ) (see Figure 2). The individual pollution variables were aggregated into daily exposure variables, at health-relevant averaging times: 24 h average for PM_2.5_ (µg/m^3^), 8 h maximum average for ground-level O_3_ (ppb), 1 h maximum for NO_2_ (ppb), and 24 h average for SO_2_ (ppb). We handled the missing data through multivariate imputation by chained equations (MICE) using predictive mean matching [28]. Guidance from in-country environmental officials aided the selection of monitoring stations that best represent daily levels in the region. Correlation coefficient cut-off values were used as inclusion criteria of monitoring stations for data imputation. The cut-point values for each pollutant are: 0.6 for PM_2.5_ and O_3_ and 0.4 for SO_2_ and NO_2_. All imputations were completed using R.

Hourly meteorological data were also obtained from REMMAQ stations and aggregated into 24 h average variables. These were used in the analysis to control for the effects of temperature and relative humidity, which have known associations with both respiratory health outcomes and daily pollution concentrations [29,30,31]. Descriptive statistics of air pollution and meteorological variable concentrations in Quito over our 2-year study period are shown in Table A1. 

The IQCA (highest daily index value from either PM_2.5_ or O_3_) is published online every day as guidance for the general population to modify their daily activities. The IQCA is a numerical scale between 0 and 500, with intermediate ranges expressed in different colors. The higher the IQCA value, the greater the level of air pollution and, consequently, the greater the health concern. The daily IQCA values for all air pollutants were calculated from daily concentrations of corresponding pollutants using equations from the technical document provided by the MDMQ (see Table 1).

### 2.2. Health Data

Hospital admission data for the years 2014–2015 for respiratory diseases in Quito were obtained from city managers, with air-pollution-relevant diagnostic codes kept in order to determine the associations of short-term pollution exposure and acute respiratory morbidity in Quito. The included diagnostic codes met the following ICD-10 definitions: acute upper respiratory infections, excluding the common cold (J01–06); pneumonia, unspecified organism (J18); other acute lower respiratory infections (J20–J22); other diseases of the upper respiratory tract (J30–J39); chronic lower respiratory disease, including COPD and asthma (J40–J47); other respiratory diseases principally affecting the interstitium (J80–J84); suppurative and necrotic conditions of the lower respiratory tract (J86); and other diseases of the pleura (J90, J92–J94). More recent years of health data through 2020 were available for analysis but were held back at the request of in-country collaborators in order to have independent data available for evaluation and validation of potential modifications to their air quality index as part of future work.

After screening with our inclusion criteria, there were a total of 19,966 respiratory hospital admissions during the study period. Daily hospital admission counts were calculated for age groups 0–17 years (children), 18–64 years (adults), 65+ years (elderly), and a combined category of all ages. The descriptive statistics by age group and year are shown in Table A2.

### 2.3. Model Design

Poisson generalized linear models were used to assess the associations of individual air pollutants with respiratory hospital admissions in Quito. Such models provide an effective method for analyzing nonlinear time-series and are widely used to analyze the health impacts of air pollution. The regression model included an indicator for day of week, a smooth function of time with four degrees of freedom (df) per calendar year to control for seasonality and long-term trends, a smooth function of same-day temperature (three df), a smooth function of lag days 1–3 temperature (three df), and a smooth function of same-day relative humidity (three df). Associations between air pollution and hospital admissions were examined for individual lag days 0–3 and average lag days 0–3. In presenting the results, excess risks and 95% confidence intervals (CI) were calculated for an interquartile range (IQR) increase in the individual air pollutants. Sensitivity analysis was completed using alternative degrees of freedom and the results indicated that the associations were not substantially changed. All analysis was completed using R [32].

The individual index values for all four air pollutants were calculated, respectively, and included in the model. The IQCA (the highest index value of the four pollutants) was also included in the model as an individual variable. The IQCA was largely driven by ozone during the 2-year study period: 575 days were driven by O_3_ and 155 days were driven by PM_2.5_. Individual index values from NO_2_ and SO_2_ were much lower than those from O_3_ and PM_2.5_, and thus were not represented in the IQCA variable.

Two-pollutant models were run to identify potential improved predictors among air pollutants. Two-pollutant models used the same basic structure as the single-pollutant models, with the inclusion of two pollutant variables with six different combinations: PM_2.5_ and O_3_, PM_2.5_ and NO_2_, PM_2.5_ and SO_2_, O_3_ and NO_2_, O_3_ and SO_2_, NO_2_ and SO_2_.

## 3. Results

**Figure 3:**
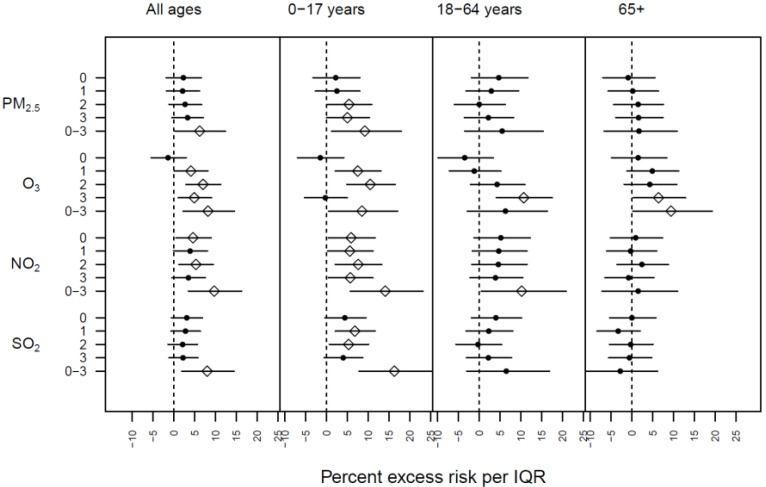
Excess risks of respiratory hospital admissions in Quito corresponding to an IQR increase in air pollutant concentrations, by lag structure and age group. Open diamonds indicate significant results and black circles indicate insignificant results at the 0.05 level.

Significant associations between air pollution exposures and daily respiratory hospital admissions were commonly observed among multiple pollutants, age groups, and lag days. Figure 3 shows the excess risks of respiratory hospital admissions in Quito, corresponding to an IQR increase in air pollutant concentrations, by lag structure and age group. Significant associations between PM_2.5_ and health outcomes were observed across multiple lag days among all ages and children, with the maximum excess risk observed on average on lag days 0–3 among children, indicating an excess risk of 9.2% (95% CI: 1.2, 18) for an IQR increase in PM_2.5_.

Exposures to increased levels of ambient O_3_ were also significantly associated with respiratory hospital admissions during the study period, with more significant associations observed for all ages compared to PM_2.5_. Similar to PM_2.5_, significant associations were mainly observed in children, but the health effects of O_3_ were also observed in older age groups with the peak impact of O_3_ occurring in adults on lag day 3 (10.7% excess risk with 95% CI: 4.1, 17.6). O_3_ was the only air pollutant that showed significant and near-significant associations with health outcomes among older adults (ages 65+).

Significant associations with respiratory hospital admissions were also observed for NO_2_. Significant associations were observed across multiple individual lag days for all ages and across all lag days in children. Among adults, there were positive but not significant associations observed across all individual lag days, and the average lag days 0–3 captured the significant associations with an excess risk of 10.2% (95% CI: 0.5, 20.9).

Associations between respiratory hospital admissions and SO_2_ were only observed in children, but the magnitude of the associations observed for average lag days 0–3 in children were the largest among all four air pollutants, with an excess risk of 16.3% (95% CI: 7.8, 25.4). No significant associations were observed among adult and elderly age groups. 

**Figure 4:**
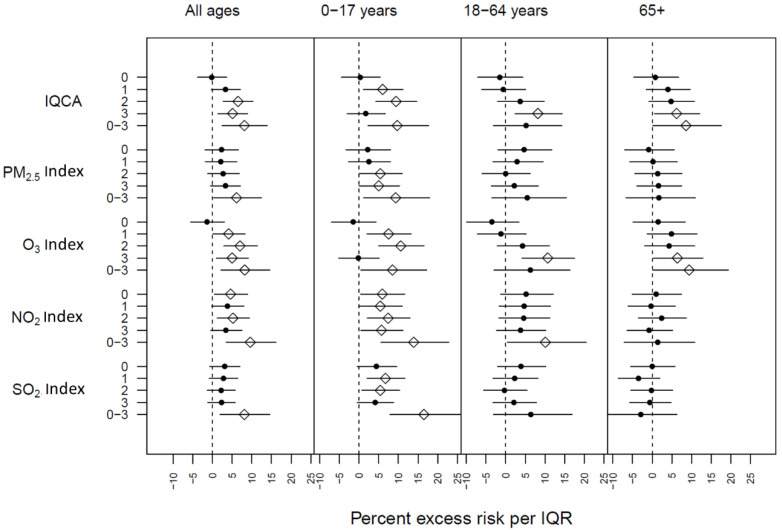
Excess risks of respiratory hospital admissions in Quito corresponding to an IQR in index values, by lag structure and age group. Open diamonds indicate significant results and black circles indicate insignificant results at the 0.05 level. IQCA is reported for the maximum daily index value across all four pollutants. Of the 730 days included in this analysis, 575 days were driven by O_3_ with the remaining 155 days driven by PM_2.5_.

Moving from individual pollutants to the local air quality index, significant associations between index values and daily respiratory hospital admissions were commonly observed among multiple pollutants, age groups, and lag days. As shown in Figure 4, associations between respiratory hospital admissions and index values followed the same pattern as their corresponding air pollutants. The effect of the daily IQCA followed a similar pattern as the O_3_ index values, which is anticipated, as most of the IQCA values came from O_3_ (575 out of 730 values). The remaining 155 IQCA values were based on the PM_2.5_ index.

**Figure A1:**
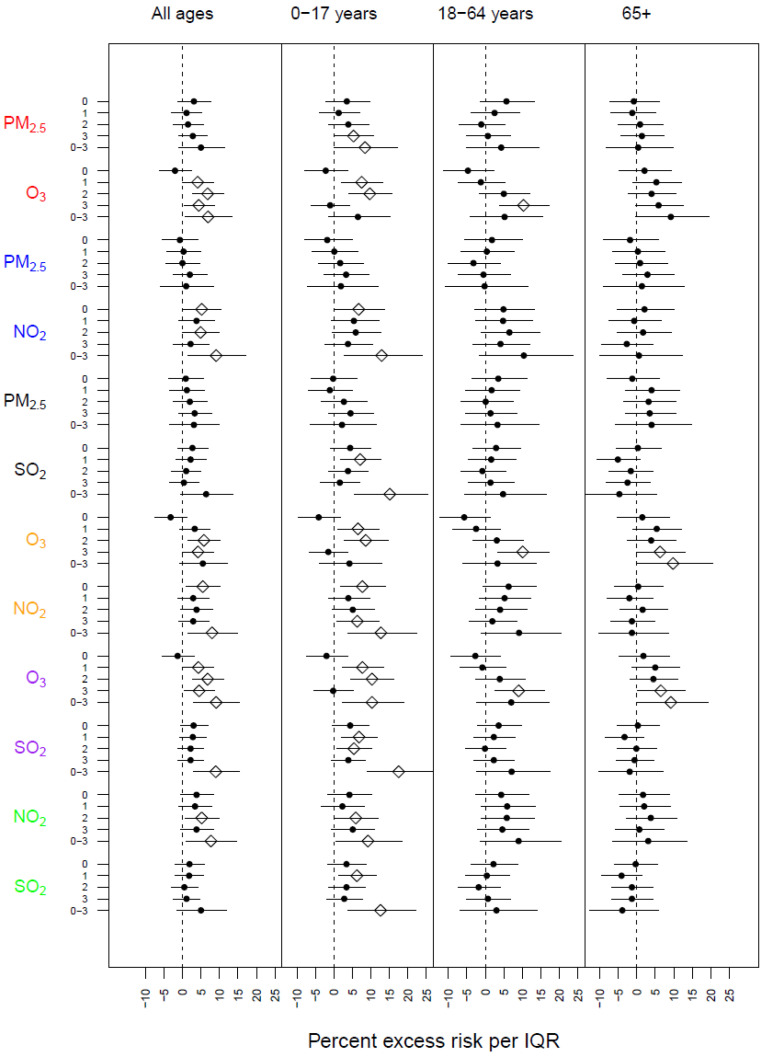
Results of two-pollutant models showing associations with daily respiratory hospital admissions by age group (six combinations grouped by color). Open diamonds indicate significant results and black circles indicate insignificant results at the 0.05 level.

The results of the two-pollutant models are presented in Figure A1. In general, the associations observed for PM_2.5_ were attenuated in most two-pollutant models, while the associations with NO_2_, and to a lesser extent O_3_, remained significant and largely unchanged, regardless of which second pollutant was also included in the analysis.

## 4. Discussion

One of the primary goals of an air quality index is to easily and effectively communicate the daily health risks of outdoor air pollution exposures to the public, especially to individuals with increased susceptibility, whom the index is designed to help. The index should take into account the effects of multiple pollutants at both high and relatively low concentrations, therein capturing the overall health risk to a population exposed to many different air pollutants.

Overall, O_3_ and NO_2_ were consistently associated with significant increases in population-level respiratory morbidity among both children and adults over multiple lag days. Average lag structures captured effects that occurred over multiple days following exposure among children. Significant results were most commonly observed among children, but this may be due in part to the higher number of hospital admissions in this age group (see Table A2) and children’s heightened susceptibility to air pollutants, as evidenced in previous studies [33,34,35]. Specifically, children have higher ventilation rates, engage in more physical activity, and spend more time outdoors than adults and thus inhale more pollutants relative to their body size. Children also have unique physiologies, including still-developing lungs, immature immune systems, and smaller peripheral airways which put them at increased risk of experiencing adverse health impacts from air pollution exposure [34,36,37,38].

The interpretation of findings of a multi-pollutant model can be complicated [39,40,41]. If the two pollutants in the same model are independent risk factors for the health outcome, a two-pollutant model might help us to capture the total impacts of these two pollutants as well as the synergistic (or antagonistic) effects. If one pollutant is a surrogate for the other, the model might be able to indicate which pollutant serves as a better predictor of the health risk, such as our two-pollutant models, which suggest that PM_2.5_ is a relatively weak predictor for health risk in Quito. However, we should be careful making such interpretations since this lower predictiveness could be caused by measurement errors or variations unique to PM_2.5_ compared to the other gaseous pollutants. If both pollutants in the model are just surrogates for some other pollutant, the model can still identify which one is a better surrogate, and thus a better predictor. Specifically, our two-pollutant model showed that NO_2_, and to a much lesser extent SO_2_, are consistently associated with health outcomes primarily among children, yet were excluded from the IQCA reporting due to their low individual index values.

This study constructed the single-pollutant index using the equations in the technical document provided by MDMQ to evaluate whether the index was associated with population-level health risks. The findings of this study showed that significant associations with respiratory morbidity were observed for all four air pollutants (PM_2.5_, O_3_, NO_2_, and SO_2_) and their IQCAs. The IQCA, which was reported online every day to the public as behavior modification guidance, was also predictive of respiratory morbidity risk among the Quito population. It is derived exclusively by O_3_ and PM_2.5_, and driven primarily by O_3_, whose pattern of effect is mimicked by the IQCA. However, only NO_2_ showed consistent significant associations with health effects in both single- and two-pollutant models among children. While NO_2_ would likely serve as a better predictor of respiratory morbidity risk than PM_2.5_ or O_3_, it is presently excluded from the IQCA reporting due to its low index values. 

An effective air quality index should provide individuals with reliable information, not just on high pollution days, but also on moderate- and low-pollution days. Cumulative evidence suggests susceptible individuals still experience adverse health risks at low levels of air pollution [13,14,42], yet commonly lack access to information that could guide their daily behavior modification decisions. An air quality index is also most useful if higher values are closely and consistently associated with increased population-level health risks. However, the nature of traditional indices only allows for a single pollutant to drive daily index values (typically the highest individual daily pollutant’s index value), potentially underestimating the total health impacts and ignoring the impacts of multi-pollutant interactions. Research in NYC has indicated that regulatory-based indices may inadequately communicate the full spectrum of adverse health risks of air pollution when there are health-relevant exposures to more than one pollutant at a time [43]. Although a traditionally designed index may be useful on its own, incorporating multiple pollutants into index calculations allows the index to better reflect the real-world health risks of air pollution.

Multi-pollutant health-based indices have been successfully implemented throughout the world. In Guangzhou, China, Li et al. (2017) constructed and validated an air quality health index based on the short-term associations of multiple air pollutants with mortality. Their findings suggest that the health-based index is a better health risk communication tool compared to the existing air quality index [20]. Cromar et al. [23] created and validated a multi-pollutant, health-based air quality index using the same three criteria pollutants in Mexico City, which is currently in use to communicate daily health risks to the public. Recently, Gladson et al. [44] developed a health-based air quality index using simple calculations based on daily index values from three criteria pollutants— PM_2.5_, NO_2_, and O_3_—which reflects children’s respiratory risk and can be used throughout the world to provide local air quality alerts. 

In light of this study’s results and the success of health-based air quality indices globally, it has been recommended that the MDMQ considers potential ways they could modify the IQCA calculation to account for the observed health impacts of all ambient air pollutants in this population. We anticipate this change will increase the health benefits of individual behavior modification influenced by Quito’s air quality alerts, especially in children. 

It is important to note that the health impacts associated with NO_2_ in particular may not be driven exclusively by its own direct health impacts. NO_2_ is a known surrogate for other traffic-related and combustion-driven air pollution that impacts health but is not typically monitored, such as ultrafine particles. These pollutants follow similar concentration patterns to NO_2_ when produced via the same processes (e.g., vehicle exhausts). The health effects of the hundreds of products of combustion are likely being reflected in the health effects linked to NO_2_ in the IQCA, as combustion is the primary source of NO_2_. In addition, because NO_2_ was the only air pollutant that showed same-day health impacts in Quito, reporting its index to the public might help people change their outdoor activities to avoid breathing polluted air on the same day. It also had a robust health effect among adults (excess risk at 10.1%, with 95% CI: 0.5 and 20.5). Furthermore, the significant associations for NO_2_ remained consistent even when controlling for a second pollutant.

There are some limitations of this study. Data were only obtained for respiratory hospital admissions, which usually have a much smaller number of daily cases compared to emergency department (ED) visits and, thus, smaller statistical power. Additionally, the data were not differentiated by at-risk populations, who may respond differently to ambient pollutant concentrations, and who may be more likely to modify their behavior based on air quality alerts.

## 5. Conclusions

This study successfully quantified the respiratory health risks associated with monitored ambient air pollution in Quito, Ecuador and identified how individual pollutants drive local risk communication. All four ambient air pollutants assessed in this study showed significant positive associations with respiratory hospital admissions, although some associations were attenuated in two-pollutant models. Quito’s risk communication tool, the IQCA, effectively represents real respiratory health risks in the region, but is driven heavily by O_3_ alone despite clear risk associations in other air pollutants, particularly NO_2_. Consideration of the impacts of all pollutants in a potential reassessment of the IQCA could help capture the overall health risk to the Quito population. This work contributes to increased global research equity by adding an epidemiological study to the limited health analyses conducted in Latin American cities.

## Figures and Tables

**Table 1:** Equations used to calculate index values based on the concentration of a given pollutant.

Contaminant (μg/m^3^)	Mathematical Expressions for Each Concentration (C) Range			
O_3_, 8 h maximum	0 < C ≤ 100	100 < C ≤ 200	200 < C ≤ 600	600 < C
	index values = C	index values = C	index values = 0.5C + 100	index values = 0.5C + 100
NO_2_, 1 h maximum	0 < C ≤ 200	200 < C ≤ 1000	1000 < C ≤ 3000	3000 < C
	index values = 0.50C	index values = 0.125C + 75.00	index values = 0.1C + 100	index values = 0.1C + 100
SO_2_, 24 h average	0 < C ≤ 62.5	62.5 < C ≤ 125	125 < C ≤ 200	200 < C
	index values = 0.8C	index values = 1.333C − 66.667	index values = 0.125C + 175	index values = 0.125C + 175
PM_2.5_, 24 h average	0 < C ≤ 50	50 < C ≤ 250	250 < C	
	index values = 2C	index values = C + 50	index values = C + 50	

## Data Availability

Health and environmental data were acquired through a special arrangement with the local government in Quito, Ecuador and are not available from the study authors for public dissemination. Requests for data should be made to the Secretaría de Ambiente in Quito.

## Appendix A

**Table A1:** Descriptive statistics of air pollution and weather data in Quito, 2014–2015. IQR: interquartile range, SD: standard deviation.

	Minimum	Median	Maximum	IQR	Mean	SD
PM_2.5_ (μg/m^3^)	6.6	17.3	32.7	6.5	17.3	4.7
O_3_ (ppb)	8.3	21.3	38.8	7.2	21.9	5.3
NO_2_ (ppb)	11.3	22	37.2	6.9	22.5	4.9
SO_2_ (ppb)	0.5	1.6	3.7	1.1	1.7	0.8
Temp (°C)	12.3	15.5	18.6	1.3	15.5	0.9
RH (%)	26.2	69.5	92.1	17.1	67.4	11.7
Precipitation (mm)	0.0	0.1	13.9	1.8	1.5	2.7

**Table A2:** Descriptive statistics of hospital admission data in Quito, 2014–2015. IQR: interquartile range, SD: standard deviation.

		Minimum Daily Count	Median Daily Count	Maximum Daily Count	IQR of Daily Count	Mean Daily Count	SD of Daily Count	Total
2014	All ages	1	26	47	11	26.3	8.1	9600
	0–17 years	0	12	25	8	12.3	5.4	4500
	18–64 years	1	8	17	5	7.9	3.8	2900
	65+	0	6	14	4	6.0	2.6	2200
2015	All ages	1	29	55	15	28.4	10.0	10,366
	0–17 years	0	10	28	6	11.0	5.0	4033
	18–64 years	0	10	24	8	10.4	5.5	3796
	65+	0	6	16	4	7.0	3.2	2537
